# Maternal and perinatal mortality by place of delivery in sub-Saharan Africa: a meta-analysis of population-based cohort studies

**DOI:** 10.1186/1471-2458-14-1014

**Published:** 2014-09-28

**Authors:** Jobiba Chinkhumba, Manuela De Allegri, Adamson S Muula, Bjarne Robberstad

**Affiliations:** University of Malawi, College of Medicine, Private Bag 360, Chichiri, Blantyre 3 Malawi; University of Bergen, Center for International Health, Bergen, Norway; Institute of Public Health, University of Heidelberg, Heidelberg, Germany

**Keywords:** Maternal and perinatal mortality risk, Place of delivery, Sub-Saharan Africa

## Abstract

**Background:**

Facility-based delivery has gained traction as a key strategy for reducing maternal and perinatal mortality in developing countries. However, robust evidence of impact of place of delivery on maternal and perinatal mortality is lacking. We aimed to estimate the risk of maternal and perinatal mortality by place of delivery in sub-Saharan Africa.

**Methods:**

We conducted a systematic review of population-based cohort studies reporting on risk of maternal or perinatal mortality at the individual level by place of delivery in sub-Saharan Africa. Newcastle-Ottawa Scale was used to assess study quality. Outcomes were summarized in pooled analyses using fixed and random effects models. We calculated attributable risk percentage reduction in mortality to estimate exposure effect. We report mortality ratios, crude odds ratios and associated 95% confidence intervals.

**Results:**

We found 9 population-based cohort studies: 6 reporting on perinatal and 3 on maternal mortality. The mean study quality score was 10 out of 15 points. Control for confounders varied between the studies. A total of 36,772 pregnancy episodes were included in the analyses. Overall, perinatal mortality is 21% higher for home compared to facility-based deliveries, but the difference is only significant when produced with a fixed effects model (OR 1.21, 95% CI: 1.02-1.46) and not when produced by a random effects model (OR 1.21, 95% CI: 0.79-1.84). Under best settings, up to 14 perinatal deaths might be averted per 1000 births if the women delivered at facilities instead of homes. We found significantly increased risk of maternal mortality for facility-based compared to home deliveries (OR 2.29, 95% CI: 1.58-3.31), precluding estimates of attributable risk fraction.

**Conclusion:**

Evaluating the impact of facility-based delivery strategy on maternal and perinatal mortality using population-based studies is complicated by selection bias and poor control of confounders. Studies that pool data at an individual level may overcome some of these problems and provide better estimates of relative effectiveness of place of delivery in the region.

**Electronic supplementary material:**

The online version of this article (doi:10.1186/1471-2458-14-1014) contains supplementary material, which is available to authorized users.

## Background

Millennium Development Goals 4 (child mortality) and 5 (maternal mortality) are inexorably linked, as the health of the mother is fundamental to the health of the newborn infant [1]. A continuum of care approach that includes prenatal, intrapartum, immediate newborn and postpartum care for mother and newborn is therefore considered essential for promotion of mother-infant health [2, 3]. Maternal and perinatal deaths are clustered around delivery [4] and the first 24 hours after birth [1] respectively. Consequently, current strategies to reduce maternal and perinatal mortality in developing countries strongly recommend that deliveries take place at health facilities compared to other settings [5]. When provided by health workers with midwifery training, facility management of deliveries might offer opportunities for early recognition of pregnancy related complications and facilitate timely provision of life saving basic and comprehensive emergency obstetric and perinatal services [6–8].

Important barriers to the supply of and demand for facility-based deliveries remain, especially among the poorest groups [9]. Key factors constraining service delivery include lack of political commitment, insufficient financial and skilled human resources and weak health care system infrastructures [10–12]. On the demand side, perceived poor quality of care, actual and opportunity cost of care seeking, cultural beliefs, lack of women empowerment and limited male involvement limit access to facility-based care [13–16]. In sub-Saharan Africa, the region with the highest maternal mortality ratio (500 deaths per 100,000 live births) and perinatal mortality rate (56 per 1,000 births) [17, 18], coverage of facility deliveries are particularly low. A recent estimate indicated that across 28 sub-Saharan countries, only 47% of births take place in a facility [19].

Over the past decade, in order to accelerate progress towards achieving MDGs 4 and 5, a number of countries in the SSA region have searched for innovative strategies to encourage women to seek care at health facilities and to increase facility-based deliveries [20, 21]. Abolition of user fees and financial incentives are some of the promising strategies. Evaluation research suggests that under certain conditions, these strategies can increase facility-based deliveries in SSA [22, 23]. However, the extent of reduction in maternal and perinatal mortality as a result of the increase in facility-based deliveries is not known.

Few studies report on the impact of place of delivery on maternal and perinatal mortality in SSA, probably reflecting the pragmatic and ethical difficulties of conducting such studies [24]. To date we are unaware of any randomized control trial (RCT), which would allow causal inference. An observational study in Nigeria has found no association between perinatal mortality and place of delivery [25]. Another study on neonatal mortality that pooled studies in low and middle income countries (LMIC) found that neonatal mortality was significantly lower for facility-based deliveries compared to home deliveries (RR 0.71, 95% CI: 0.54-0.87), but this did not include stillbirths or maternal outcomes [26]. Robust evidence on the relative effectiveness of place of delivery, using health outcome measures, is needed to inform policy formulation in SSA. This information may also make it possible to assess the comparative effectiveness of alternative interventions for reducing maternal and perinatal mortality.

The aim of this paper is to estimate from secondary data how maternal and perinatal mortality in SSA is affected by place of delivery. Mortality is chosen as a health outcome because it is relatively easy to measure and therefore likely to achieve valid results [27]. Additionally, unlike neonatal mortality that only takes live births into account, perinatal mortality also includes stillbirths making it a comprehensive and suitable indicator for assessing outcomes of both intrapartum and immediate post partum care services [28].

## Methods

### Literature search

Using a predefined protocol, we carried out a systematic search of the literature following guidelines for meta-analysis of observational studies in Epidemiology (MOOSE) [29] and preferred reporting items for systematic reviews and meta-analysis (PRISMA) (see Additional file 1). The search was conducted between January and August 2013. A physician with support of a librarian conducted the search. Medical and social science databases and journal libraries included, but were not limited to, PubMed, EBSCO Host, Web of Science, ScienceDirect, Wiley, Cochran library and Google Scholar. We searched for studies conducted in SSA, involving pregnant women and reporting on risk of maternal and perinatal mortality/death by place of birth or delivery. Reference lists of selected publications were assessed in order to identify other potential papers of interest.

The key words used were “Maternal mortality”, “Maternal deaths”, “Perinatal mortality”, “Perinatal deaths”, risk, “Place of birth/delivery”, study, Africa and “sub-Saharan Africa”. In the selected publications, the following definitions were used. Maternal deaths were defined as all direct and indirect obstetric deaths during pregnancy, delivery, and the first 42 days after birth. Perinatal deaths were defined as pregnancy losses occurring after seven completed months of gestation (stillbirths), or deaths within the first seven days of delivery of a live born child (early neonatal deaths) weighing 1000 grams or more. Place of delivery was either facility-based (defined as birth or delivery in a formal health facility whether or not attended by a skilled medical attendant) or home (defined as birth or delivery outside of a formal health facility whether or not attended by a skilled traditional birth attendant). Studies reporting on perinatal mortality were considered eligible if they reported on pregnancy outcomes from 7 complete months until 7 days after birth. Studies reporting on maternal mortality were eligible if they reported on maternal deaths as soon as pregnancy was identified until 42 days after birth. In addition the studies had to be written in English and published between 1990 and 2013.

### Data quality and extraction

Each study was subjected to a quality review using a modified Newcastle-Ottawa scale for cohort studies [30]. Key quality items assessed included representativeness of the population, population characteristics such as gestation age at enrollment and duration of pregnancy follow up, information about study design (population based cohort vs. demographic and health surveillance), ascertainment of exposure and proportion of home deliveries, and use of standard definitions for main outcome measures and denominators used (e.g. births, live births). We also extracted information that may have affected the outcomes such as frequency of data collection, proportion of refusals, loss to follow up and sample sizes.

We noted general study information (e.g. year of study publication and authors) and the primary health outcomes for the study: maternal and perinatal mortality ratio by place of delivery. As the outcomes of interest are ratios, we extracted information on relevant numerators and denominators that would enable independent calculations of these ratios. In cases where an appropriate denominator was not provided (e.g. number of live births at home), but the numerator and corresponding appropriate ratio was given, we estimated the denominator by simple proportion. We precluded studies that aimed to ascertain risk of a particular exposure (e.g. HIV infection or severe anaemia in pregnancy) or an intervention (e.g. Prevention of mother to child transmission of HIV) on perinatal/maternal mortality, with insufficient (<25%) assignment of outcomes of interest to place of delivery and where reported data could not completely fill a 2 × 2 table. Where possible, we contacted some authors either to confirm information or provide extra details. Two individuals independently extracted the information and resolved disparities by consensus.

### Statistical analysis

Study quality was scored in two categories: high if more than 60% of the quality items were reported and low otherwise. For both primary health outcomes of interest, we estimated the crude odds ratio (OR) by place of delivery in each study and then calculated the weighted average of the OR across the studies using meta analytic procedures [31]. The OR from each study can be combined using a variety of analytical methods, which are classified as: a) fixed effect models which weight studies according to the amount of information they contain; or b) random-effects models, which incorporate an estimate of between-study variation in the weighting [32, 33]. We assessed study heterogeneity using I^2^ statistic, which measures the percentage of variation in OR attributable to heterogeneity between studies. The fixed effects model was used when I^2^ was low < 50% [34] otherwise we used random-effects model to calculate individual study OR and corresponding 95% confidence intervals (95% CI) and to pool the results across the studies.

The potential effect of place of delivery on maternal and perinatal mortality was estimated by attributable risk percentage reduction, defined as the portion of the incidence of an outcome in the exposed that is due to the exposure: [(Ie-Iu)/Ie]*100 where Ie and Iu are incidences in the exposed and unexposed groups, respectively [35]. In our analysis, this represents the incidence of mortality in the exposed (home delivery group) that would be prevented if the exposure (home deliveries) were eliminated. We used a Poisson method to calculate mortality ratios and their 95% CIs as Poisson method approximates distribution of these ratios better [36]. STATA version 12.0 (Stata Corp, College Station, Texas) was used for analysis. No ethical review was required for the study.

## Results

**Figure 1:**
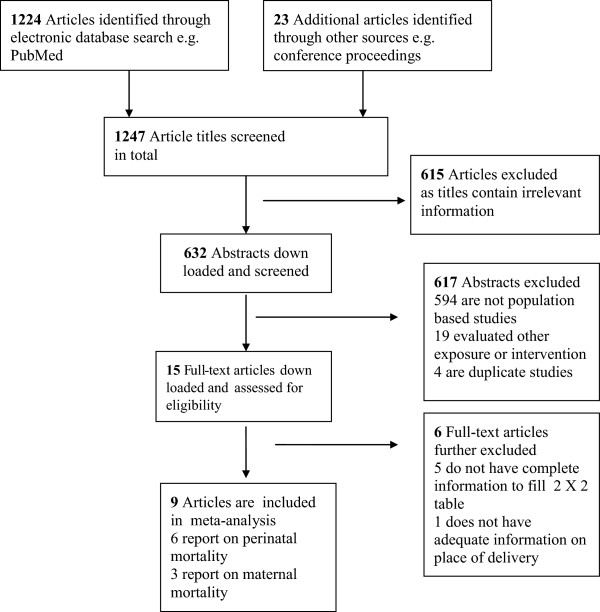
**Flow chart showing identification, screening and inclusion of studies for meta-analysis of maternal and perinatal mortality by place of delivery in sub-Saharan Africa, 1990–2013.**

As shown in Figure 1, a total of 1247 studies were identified through the literature search. We discarded 615 studies after appraising the titles as they contained irrelevant information or were redundant. We then screened abstracts for the remaining 632 studies before excluding 617 more studies. The reasons for exclusions were a) the studies were not population based or did not report outcomes by place of delivery (n = 594); b) the studies were evaluating impact of an interventions or risk of an exposure (n = 19); and c) the studies were duplicate publications (n = 4). We retrieved 15 studies in full, of which 6 studies were further excluded (5 prospective cohorts and 1 retrospective cohort) because the reported data for 5 studies were not sufficient to completely fill a 2 × 2 table [37–41] and 1 study [42] did not have enough information on place of delivery.

Of the 9 studies that met the inclusion criteria for the analysis: 6 studies [43–48] reported on perinatal mortality and the other 3 studies [36, 49, 50] reported on maternal mortality. All were population based prospective cohort studies and had high quality scores. The mean study quality score for the selected studies was 10 out of 15 possible points (see Additional file 2). The most common limitations identified were lack of randomization in group allocation and lack of independent blind assessment of study outcomes. The 9 retained studies contained information on 36,772 pregnancy episodes in total. Of these 9,362 (25.5%) had information on the perinatal outcome alone. Table 1 provides further details on the characteristics of the studies included in the meta-analysis.

**Table 1:** **Characteristics of studies included in meta-analysis of perinatal and maternal mortality by place of delivery in sub-Saharan Africa**

Author (s)	Year of publication	Study country	Study setting	Study design	Home births (%)	Refusals (%)	Lost follow ups (%)	Sample size	Deaths/Births (n/N)	
									Facility	Home
*Perinatal studies*										
Walraven et al.	1995 [43]	Tanzania	Rural	Prospective cohort	52.7	NP	3.8	447	7/202	22/225
McDermott et al.	1996 [45]	Malawi	Rural	Prospective cohort	41.6	NP	4.3	4,052	131/2257	133/1609
Diallo et al.	2010 [46]	Burkina Faso	Rural	Prospective cohort	64.4	1.8	<0.1	900	26/326	46/589
Nankambirwa et al.	2011 [44]	Uganda	Rural	Prospective cohort	41.5	1.0	3.0	835	13/490	21/347
Matendo et al.	2011 [52]	DRC*	Rural	Prospective cohort	78.3	1.0	<0.1	1,886	34/411	82/1481
Schmiegelow et al.	2012 [47]	Tanzania	Rural	Prospective cohort	16.7	1.1	3.9	995	41/726	5/146
*Maternal studies*										
De Bernis et al.	2000 [49]	Senegal	Urban	Prospective cohort	57.4	<0.1	<0.1	3,777	12/2160	4/1316
Bouvier-Colle et al.	2001 [36]	West Africa**	Rural/Urban	Prospective cohort	26.4	NP	<0.1	20,326	50/10058	5/3621
Høj et al.	2002 [50]	Guinea-Bissau	Rural	Prospective cohort	75.4	NP	NP	14,257	35/2489	50/7610

*Democratic republic of Congo **Countries included: Ivory Cost, Mali, Niger, Mauritania, Burkina Faso and Senegal. NP-Not provided.

### Perinatal mortality

**Figure 2:**
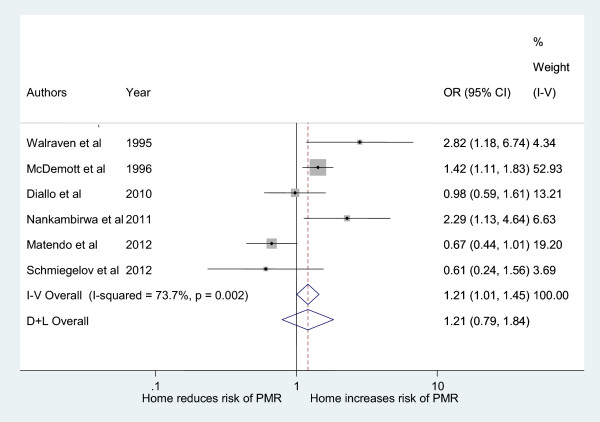
**Pooled analysis of perinatal mortality by place of delivery in sub-Saharan Africa.**

To estimate the protective effect of place of delivery, we first calculated the odds of perinatal mortality by place of delivery. Using a fixed effects model, Figure 2 shows that the pooled crude odds of perinatal mortality is significantly higher for home compared to facility delivery (OR 1.21, 95% CI: 1.02-1.46). As there is a high between-study heterogeneity I^2^ = 73.7, we also estimated the pooled effect with a random effects model [51]. The estimate from the more conservative random effects model is exactly the same, but is no longer significant, (OR 1.21, 95% CI: 0.79-1.84). The results of the individual studies are also mixed. Two studies are in favor of home delivery [47, 52], one is neutral [46] and three are in favor of facility-based delivery [43–45].

Next, we estimated the actual perinatal mortality ratio (PMR) by place of delivery. Table 2 shows that the overall weighted PMR is 63 (95% CI: 54**–**73) per 1000 births. The PMR is 70 (95% CI: 57–86) and 56 (95% CI: 44–69) per 1000 births for home and facility-based deliveries, respectively. The attributable risk percentage is 21% (95% CI:-6,40).

**Table 2:** **Weighted perinatal and maternal mortality ratios by place of delivery in sub-Saharan Africa**

Outcomes/place of birth	Dead	Alive	Mortality ratio (95% CI)*	Attributable% (95% CI)*
*Perinatal mortality*				
Home deliveries	95	1,258	70 (57–86)	21 (-6,40)
Facility deliveries	82	1,387	56 (44–69)	
***Total***	**177**	**2,645**	**63 (54–73)**	
*Maternal mortality*				
Home deliveries	38	6,302	599 (424–823)	N/A
Facility deliveries	35	3,668	945 (658–1315)	
***Total***	**73**	**9,970**	**726 (570.–913)**	

*Estimated using Poisson exact method. N/A –Not applicable.

### Maternal mortality

**Figure 3:**
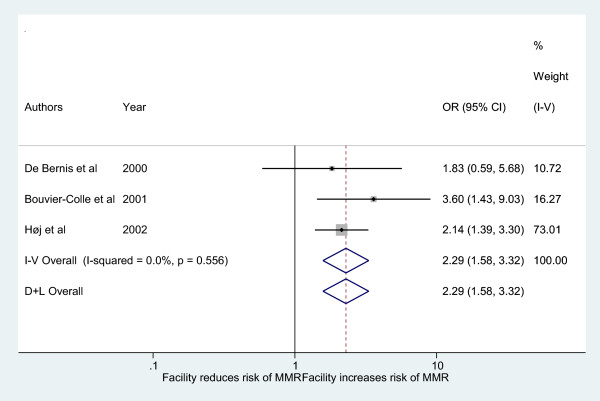
**Pooled analysis of maternal mortality by place of delivery in sub-Saharan Africa.**

Figure 3 shows that the estimated pooled crude odds for maternal mortality at facility is 2.29 (95% CI: 1.58-3.31) compared to home settings. There is no variation in OR attributable to heterogeneity, I-squared = 0%. The increased odds for maternal mortality at facility are consistently high across all the three individual maternal studies and stable across both fixed and random effect models. The overall weighted maternal mortality ratio (MMR) is 727 (95% CI: 570–913) deaths per 100, 000 live births. The MMR is 599 (95% CI: 424–823) and 945 (95% CI: 658–1315) per 100,000 live births for home and facility deliveries, respectively (Table 2).

## Discussion

### Perinatal mortality

In this study, we attempted to estimate the risk of mortality for home deliveries compared to facility-based deliveries. The study illustrates the complexity of the relationship between risk of mortality and place of delivery. At an individual level, we found evidence for increased chance of perinatal losses following home compared to facility-based deliveries. However, opposite conclusions can statistically be reached, significant versus non significant results, depending on whether we make fixed model or random effect model assumptions. It has been argued that a finding that is statistically significant with the latter, but not with the former model, should be viewed with caution [51, 53].

We estimated the overall weighted perinatal mortality ratio in SSA to be 63 (95% CI: 54–73) deaths per 1000 births. This figure compares well with current WHO estimates of PMR (56 per 1000 births) in the region [18]. Our results imply that at best, an expected 14 perinatal deaths could be averted per 1000 births if women delivered at facility instead of home. This represents a 21% (95% CI:-6,40) reduction in perinatal mortality risk for the home delivery group. In 2012, 36.8 million births were estimated to have taken place in SSA of which 18.0 million (49%) were home deliveries [54]. In view of the high number of home deliveries in the SSA region, it can be argued that a 21% reduction in perinatal mortality risk might produce important public health improvements, if only a significant portion of the women who currently give birth at home could be motivated to deliver in health facilities.

Through health worker trainings and WHO policy [5], the current practice in the region is to encourage active or self referral of pregnant women, especially those with high risk factors (e.g. twin pregnancy, pregnancy induced hypertension) to deliver at health facilities. Moreover, some of the studies included in the analysis made special arrangements for facility referral of at risk pregnancies [43, 47]. This selection was likely to have made the facility and home delivery groups different with respect to perinatal risk factors, the latter being on average less at risk than the former. The results might therefore represent an underestimation of PMR in the home delivery group. The observation that perinatal mortality is on average high in the home delivery group, in spite of its relative low risk profile, should be of concern to policy makers, program implementers and health care providers in SSA.

### Maternal mortality

Regarding maternal mortality, the results show that pregnant mothers delivering in facilities have a significantly higher risk of experiencing a maternal death than women delivering at home. Several reasons could account for this surprising result. First, in settings where access to facility-based delivery is low (<40%), those seeking care at facilities may be complicated cases with higher risk of mortality [55, 56]. In such settings, high maternal mortality at facilities would be expected due to high risk selection, as already reported in the literature [57]. Only one [36] out of the three studies reporting on maternal mortality had a low facility delivery rate. Precluding this study from the analysis does not significantly change the results. This suggests that low facility delivery rate alone does not appear to explain the relatively high risk of mortality observed for facility-based deliveries in this study. Second, and related to the risk selection, the study by Høj et al. [50] shows a progressive increase in risk of maternal mortality from health center to hospital compared to home: (OR 1.49,95% CI: 0.73-2.76) and (OR 2.72, 95% CI:1.64-4.38) respectively. This suggests existence of a differential in terms of the complexity of case mix by level of care. This can be interpreted as an indication that referral for facility-based delivery is actually working with secondary or tertiary referral facilities treating women with more complex conditions [36], although probably the timeliness of care is not optimal to make a difference [58]. Thirdly, facility-based delivery as a strategy to reduce maternal mortality does not simply entail delivery at a health facility, but also access to an enabling environment, such as availability of health workers with midwifery training, diagnostic tools, drug supplies and access to blood bank for effective care. High risk of maternal mortality at facility may therefore reflect lack of requisite capacity for facilities to offer quality care to high-risk women [59]. A large study in the region has shown that most maternal deaths occurring at facilities are among women who receive substandard care [36]. Moreover, not all facilities-based deliveries are attended by health workers with midwifery training due to unfilled vacancies and staff absenteeism [60], supporting the assertion that poor quality of care at health facilities is likely to explain a significant portion of the observed high risk of facility-based maternal deaths. Fourthly, although hard evidence is lacking, the possibility that mothers might be harmed at facilities, due for example to poor infection control or other human errors, cannot be ruled out [55].

### Strengths and limitations

The strength of our paper accrues from the deliberate choice to include studies with a homogenous design, using standard definitions of outcomes (perinatal and maternal mortality ratios), and that largely collected data prospectively and at regular intervals to minimize recall bias and increase validity of the outcome measures. Typically, estimates of perinatal and maternal mortality in African countries are derived from complex statistical modeling techniques [48] or from nationally representative demographic and health surveys that use sisterhood approach methods [61]. However, poor quality data, recall bias and selective under reporting tend to affect such estimates [25].

Still, our study suffers from some limitations. Population-based cohort studies are costly and therefore only few have been conducted in the SSA region. Despite the high quality of individual eligible studies, the pooled analysis is based on a limited number of studies, thus the results should be interpreted with caution. We also noted subtle operational variations in the exposures. For example, home delivery in some settings meant delivery by non-trained traditional birth attendants (TBAs) [43] while in other settings it entailed delivery by trained TBAs [52]. Despite the uniform study design, such conceptual differences can lead to clinical variations [32] and heterogeneity.

Geographical, temporal, health systems and individual patient risk profiles can also affect maternal and perinatal outcomes [58] and confound interpretation of health outcomes by place of delivery. It is known for instance that rural areas tend to have worse perinatal and maternal health outcomes than urban areas [4]. Within the SSA region, important variations in the risk of mortality exist between sub-regions [62]. At an individual level, age, parity and existence of other co-morbidities such as hypertension can influence both maternal and perinatal outcomes [47, 50]. Ideally, these factors should be controlled before making statements about the relative effectiveness of one place of delivery compared to another [55]. Inadequate number of studies and particularly the lack of patient level information from the available studies precluded the possibility of such accurate analysis in our own review.

## Conclusions

Maternal mortality ratio has always been difficult to estimate because maternal deaths are relatively rare events [63]. The observed high risk for maternal deaths at facilities makes it problematic to use this outcome measure to assess the potential impact of interventions that promote facility-based deliveries. The impact of interventions aimed at increasing facility-based deliveries may therefore be better estimated in relation to reductions in morbidity, not just mortality. Studies of maternal illness such as severe maternal complications, which occur in far greater numbers than maternal deaths, may instead allow for robust quantification and evaluation of interventions that promote facility-based deliveries and have been suggested as alternatives to assessment of maternal mortality [64].

Put together, our results appear to suggest that as a strategy to reduce maternal and perinatal mortality, facility-based delivery is more likely to reduce perinatal than maternal mortality. Interventions that promote facility-based deliveries may likely contribute more to attainment of MDG4 (child mortality) than MDG 5(maternal mortality) in the SSA region. Current evidence of poor quality of care and high risk of maternal mortality at facilities emphasis the need for quality improvement efforts to precede activities aimed at increasing demand for facility-based deliveries in the SSA region [65].

Evaluating the impact of facility-based delivery strategy on maternal and perinatal mortality using population-based studies is complicated by selection bias in favor of women that deliver at facilities and poor control of confounders. Studies that pool data at an individual level may allow for better control of confounding/risk modifying factors and provide better estimates of relative safety of places of delivery in the region. Future studies should focus on assessing the relative contribution of poor quality and delayed care seeking on facility based maternal and perinatal deaths to better prioritize resources and align interventions efforts.

## Electronic supplementary material

Additional file 1: **PRISMA Checklist_maternal and perinatal mortality by place of delivery SSA.** (DOC 64 KB)

Additional file 2: **Assessment of studies used in analysis of maternal and perinatal mortality against elements of good quality cohort design.** (DOC 43 KB)

## References

[1] Filippi V, Ronsmans C, Campbell OMR, Graham WJ, Mills A, Borghi J, Koblinsky M, Osrin D (2006). Maternal health in poor countries: the broader context and a call for action. Lancet.

[2] Bhutta ZA, Darmstadt GL, Hasan BS, Haws RA (2005). Community-Based Interventions for Improving Perinatal and Neonatal Health Outcomes in Developing Countries: A Review of the Evidence. Pediatrics.

[3] Kerber JK, de Graft-Johnson EJ, Bhutta AZ, Okong P, Starrs A, Lawn J (2007). Continuum of care for maternal, neonatal, and child health: from slogan to service delivery. Lancet.

[4] Ronsmans C, Graham WJ (2006). Maternal mortality: who, when, where, and why. Lancet.

[5] WHO (2005). The World Health Report 2005: make every mother and child count.

[6] United Nations (2008). Millennium Development Goals Report.

[7] Starrs A (2007). Delivering for Women. Lancet.

[8] Paxton A, Maine D, Freddman L, Fry D, Lobis S (2005). The evidence for emergency obstetric care. Int J Gynaecol Obstet.

[9] Montagu D, Yarney G, Visconti A, Harding A, Yoong J (2011). Where do poor women in developing countries give birth? A multi-country analysis of demographic and health survey data. PLoS One.

[10] Prata N, Passano P, Sreenivas A, Gerdts CE (2010). Maternal mortality in developing countries: challenges in scaling-up priority interventions. Womens Health (Lond Engl).

[11] Jacobs B, Ir P, Bigdeli M, Annear PL, Van DW (2012). Addressing access barriers to health services: an analytical framework for selecting appropriate interventions in low-income Asian countries. Health Policy Plan.

[12] Prata N, Passano P, Rowen T, Bell S, Walsh J, Potts M (2011). Where there are (few) skilled attendants. J Health Popul Nutr.

[13] McNamee P, Ternent L, Hussein J (2009). Barriers in accessing maternal healthcare: evidence from low and middle-income countries. Expert Rev Pharmacoecon Outcomes Res.

[14] Parkhurst JO, Rahman SA, Ssengooba F (2006). Overcoming access barriers for facility-based delivery in low-income settings: insights from Bangladesh and Uganda. J Health Popul Nutr.

[15] Diaz-Granados N, Pitzul KB, Dorado LM, Wang F, McDermott S, Rondon MB, Posada-Villa J, Saavedra J, Torres Y, Des Meules M, Stewart DE (2011). Monitoring gender equity in health using gender-sensitive indicators: A cross-national study. J Women’s Health.

[16] Borghi J, Ensor T, Somanathan A, Lissner C, Mills A (2006). Mobilizing financial resources for maternal health. Lancet.

[17] WHO (2012). Trends in Maternal Mortality: 1990 to 2010. WHO, UNICEF, UNFPA and The World Bank estimates.

[18] WHO (2007). Neonatal and perinatal mortality, 2004: country, regional and global estimates.

[19] Kruk ME, Paczkowski M, Mbaruku G, de Pinho H, Galea S (2009). Women's Preferences for Place of Delivery in Rural Tanzania: A Population-Based Discrete Choice Experiment. Am J Public Health.

[20] Gorter AC, Ir P, Meessen B (2013). Evidence Review, Results-Based Financing of Maternal and Newborn Health Care in Low- and Lower-middle-Income Countries, February 2013, study commissioned and funded by the German Federal Ministry for Economic Cooperation and Development (BMZ) through the sector project PROFILE at GIZ – Deutsche Gesellschaft für Internationale Zusammenarbeit.

[21] Meessen B, Soucat A, Sekabaraga C (2011). Performance-based financing: just a donor fad or a catalyst towards comprehensive health-care reform?. Bull World Health Organ.

[22] De Allegri M, Ridde V, Louis VR, Sarker M, Tiendrebéogo J, Yé M, Müller O, Jahn A (2011). Determinants of utilization of maternal care services after the reduction of user fees: a case study from rural Burkina Faso. Health Policy Plan.

[23] Basinga P, Gertler PJ, Binagwaho A, Soucat AL, Sturdy J, Vermeersch CM (2011). Effect on maternal and child health services in Rwanda of payment to primary health-care providers for performance: an impact evaluation. Lancet.

[24] Nove A, Berrington A, Matthews Z (2012). The methodological challenges of attempting to compare the safety of home and hospital birth in terms of the risk of perinatal death. Midwifery.

[25] Otia SO, Odimegwu C (2011). Perinatal Mortality in Nigeria: Do Place of Delivery and Delivery Assistants Matter?. Open Demography J.

[26] Tura G, Fantahun M, Worku A (2013). The effect of health facility delivery on neonatal mortality: systematic review and meta-analysis. BMC Pregnancy Childbirth.

[27] Anker M, Black RE, Coldham C, Kalter HD, Quigley M, Ross D, Snow RW (1999). A Standard Verbal Autopsy Method for Investigating Causes of Death in Infants and Children.

[28] Darmstadt GL, Yakoob MY, Haws RA, Menezes EV, Soomro T, Bhutta ZA (2009). Reducing stillbirths: interventions during labour. BMC Pregnancy Childbirth.

[29] Stroup DF, Berlin JA, Morton SC, Ingram Olkin G, Williamson D, Rennie D, Moher D, Becker BJ, Sipe TA, Thacker SB (2000). Meta-analysis of Observational Studies in Epidemiology A Proposal for Reporting. JAMA.

[30] Wells GA, Shea B, O'Connell D, Peterson J, Welch V, Losos M, Tugwell P: **The Newcastle-Ottawa Scale (NOS) for assessing the quality of nonrandomised studies in meta-analyses.** http://www.ohri.ca/programs/clinical_epidemiology/oxford.asp (Last accessed on 30 November, 2013)

[31] Kirkwood BR, Sterne JAC (2003). Essential Medical Statistics.

[32] Haidich AB (2010). Meta-analysis in medical research. HIPPOKRATIA.

[33] Harris RJ, Bradburn MJ, Deeks JJ, Harbord RM, Altman DG, Sterne JAC (2008). metan: fixed- and random-effects meta-analysis. The Stata Journal 8.

[34] Higgins JPT, Thompson SG, Deeks JJ, Altman DG (2003). Measuring inconsistency in meta-analyses. Br Med J.

[35] Daly LE, Geoffrey J (2007). Bourke Interpretation and Uses of Medical Statistics.

[36] Bouvier-Colle MH, Ouedraogo C, Dumont A, Vangeenderhuysen C, Salanave B, Decam C, MOMA group (2001). Maternal mortality in West Africa. Rates, causes and substandard care from a prospective survey. Acta Obstet Gynecol Scand.

[37] Aisien AO, Lawson JO, Okolo A (2000). Two years prospective study of perinatal mortality in Jos, Nigeria. Int J Gynecol Obstet.

[38] Chalumeau M, Salanave B, Bouvier-Colle MH, de Bernis L, Prua A, Bréart G (2000). Risk factors for perinatal mortality in West Africa: a population-based study of 20 326 pregnancies. MOMA group. Acta Paediatrica.

[39] Paul W, Moyer CA, Raymond A, Philip A, John W, Welaga P, Moyer CA, Aborigo R, Adongo P, Williams J, Hodgson A, Oduro A, Engmann C (2013). Why Are Babies Dying in the First Month after Birth? A 7- Year Study of Neonatal Mortality in Northern Ghana. PLoS ONE.

[40] Lawoyin TO, Onadeko MO, Asekun-Olarinmoye EO (2010). Neonatal Mortality and Perinatal Risk Factors in Rural Southwestern Nigeria: A Community-Based Prospective Study. W Afr J Med.

[41] Kulmala T, Vaahtera M, Rannikko J, Ndekha MD, Cullinan T, Salin ML, Ashorn P (2000). The relationship between antenatal risk characteristics, place of delivery and adverse delivery outcome in rural Malawi. Acta Obstet Gynecol Scand.

[42] Engmann C, Walega P, Aborigo RA, Adongo P, Moyer CA, Lavasani L, Williams J, Bose C, Binka F, Hodgson A (2012). Stillbirths and early neonatal mortality in rural Northern Ghana. Trop Med Int Health.

[43] Walraven GE, Mkanje RJ, Roosmalen J, van Dongen PW, Dolmans WM (1995). Perinatal mortality in home births in rural Tanzania. Eur J Obstet Gynecol Reprod Biol.

[44] Nankabirwa V, Tumwine JK, Tylleskär T, Nankunda J, Sommerfelt H, PROMISE EBF Research Consortium (2011). Perinatal mortality in eastern Uganda: a community based prospective cohort study. PLoS One.

[45] McDermott J, Steketee VR, Wirima J (1996). Perinatal mortality in rural Malawi. Bull World Health Organ.

[46] Diallo AH, Meda N, Zabsonré E, Sommerfelt H, Cousens S, Tylleskär T, PROMISE-EBF Study Group (2010). Perinatal mortality in rural Burkina Faso: a prospective community-based cohort study. BMC Pregnancy Childbirth.

[47] Schmiegelow C, Minja D, Oesterholt M, Pehrson C, Suhrs HE, Boström S, Lemnge M, Magistrado P, Rasch V, Lusingu J, Theander TG, Bruun Nielsen B (2012). Factors associated with and causes of perinatal mortality in northeastern Tanzania. Acta Obstet Gynecol Scand.

[48] Stanton C, Lawn JE, Rahman H, Wilczynska-Ketende K, Hill K (2006). Stillbirth rates: delivering estimates in 190 countries. Lancet.

[49] De Bernis L, Dumont A, Bouillin D, Gueye A, Dompnier PJ, Bouvier-Colle MH (2000). Maternal morbidity and mortality in two different populations of Senegal: a prospective study (MOMA survey). BJOG.

[50] Høj L, da Silva D, Hedegaard K, Sandstro¨m A, Aaby P (2002). Factors associated with maternal mortality in rural Guinea-Bissau. A longitudinal population-based study. BJOG.

[51] DerSimonian R, Laird N (1986). Meta-analysis in clinical trials. Control Clin Trials.

[52] Matendo MR, Engmann MC, Ditekemena DJ, Gado J, Tshefu A, McClure EM, Moore J, Boelaert M, Carlo WA, Wright LL, Bose CL (2011). Challenge of Reducing Perinatal Mortality in Rural Congo: Findings of a Prospective, Population-based Study. J Health Popul Nutr.

[53] Garg AX, Dan H, Marcello T (2008). Systematic Review and Meta-analysis: When One Study Is Just not Enough. Clin J Am Soc Nephrol.

[54] Singh S, Darroch JE, Ashford LS (2013). Adding It Up: The Need for and Cost of Maternal and New- born Care—Estimates for 2012.

[55] Lohela TJ, Campbell OMR, Gabrysch S (2012). Distance to Care, Facility Delivery and Early Neonatal Mortality in Malawi and Zambia. PLoS ONE.

[56] Mcclure EM, Goldenburg RL, Bann CM (2007). Maternal mortality, stillbirth and measures of obstetric care in developing and developed countries. Int J Gynaecol Obstet.

[57] Ronsmans C, Chowdhury ME, Koblinsky M, Ahmed A (2010). Care seeking at time of childbirth, and maternal and perinatal mortality in Matlab, Bangladesh. Bull World Health Organ.

[58] Scott S, Ronsmans C (2009). The relationship between birth with a health professional and maternal mortality in observational studies: a review of the literature. Trop Med Int Health.

[59] Blum LS, Sharmin T, Ronsmans C (2006). Attending home vs. clinic-based deliveries: perspectives of skilled birth attendants in Matlab, Bangladesh. Reproductive Health Matters.

[60] Mueller DH, Lungu D, Acharya A, Palmer N (2011). Constraints to implementing the Essential Health Package in Malawi. PloS one.

[61] Lawn JE, Cousens S, Zupan J (2005). 4 million neonatal deaths: when? Where? Why?. Lancet.

[62] Moyer CA, Dako-Gyeke P, Adanu RM (2013). Facility-based Delivery and Maternal and Early Neonatal Mortality in Sub-Saharan Africa: A Regional Review of the Literature. Afr J Reprod Health.

[63] Graham WJ, Filippi VGA, Ronsmans C (1996). Demonstrating programme impact on maternal mortality. Health Policy Plan.

[64] Adeoye AI, Onayade AA, Fatusi OA (2013). Incidence, determinants and perinatal outcomes of near miss maternal morbidity in Ile-Ife Nigeria: a prospective case control study. BMC Pregnancy Childbirth.

[65] Maine D, Rosenfield A (1999). The Safe Motherhood Initiative: why has it stalled?. Am J Public Health.

[CR66] The pre-publication history for this paper can be accessed here:http://www.biomedcentral.com/1471-2458/14/1014/prepub

