# Supplementary material for: Maternal and perinatal mortality by place of delivery in sub-Saharan Africa: a meta-analysis of population-based cohort studies
Source: BMC Public Health. 2014 Sep 28;14:1014. doi: 10.1186/1471-2458-14-1014 (PMC4194414; doi:10.1186/1471-2458-14-1014)
Supplement: Supplementary file 2 — Additional file 2: Assessment of studies used in analysis of maternal and perinatal mortality against elements of good quality cohort design. (DOC 43 KB) [file 12889_2014_7129_MOESM2_ESM.doc]

**Assessment of studies used in analysis of maternal and perinatal mortality against elements of good quality cohort design**

|  |  | ***Perinatal studies*** | | | | | | ***Maternal studies*** | | |
| --- | --- | --- | --- | --- | --- | --- | --- | --- | --- | --- |
| ***Quality Items*** | **Author**  **Score#** | Walraven  et al[43] | McDermott  et al[45] | Diallo  et al[46] | Nankambirwa  et al[44] | Matendo  et al[52] | Schmiegelow  et al[47] | De Bernis  et al[49] | Bouvier-Colle  et al[36] | Høj  et al[50] |
| Exposed cohort representative of community | 1 | Yes | Partly | Yes | Yes | Yes | Yes | Yes | Yes | Yes |
| Non exposed cohort from same community | 1 | Yes | Partly | Yes | Yes | Yes | Yes | Yes | Yes | Yes |
| Random allocation of exposure | 2 | No | No | No | No | No | No | No | No | No |
| Exclusion of outcome of interest at start of study | 1 | Yes | Yes | Yes | Yes | Yes | Yes | Yes | Yes | Yes |
| Sample size calculations | 2 | No | No | No | No | No | No | No | No | No |
| Adequate sample size | 1 | Partly | Yes | Yes | Yes | Yes | Yes | Yes | Yes | Yes |
| Adjustment for confounding | 2 | Yes | Yes | Yes | Yes | Yes | Yes | No | No | Yes |
| Objective outcome | 2 | Yes | Yes | Yes | Yes | Yes | Yes | Yes | Yes | Yes |
| Rigour of outcome assessment | 1 | Yes | Yes | Yes | Yes | Yes | Yes | Yes | Yes | Yes |
| Adequate follow up for outcomes to occur | 1 | Yes | Yes | Yes | Yes | Yes | Yes | Yes | Yes | Yes |
| Low loss to follow up of cohort | 1 | Yes | Yes | Yes | Yes | Yes | Yes | Yes | Yes | Unclear |
| ***Total quality score*** | 15 | 10 | 9 | 11 | 11 | 11 | 11 | 10 | 10 | 9 |

#: Maximum score for each quality item.
